# Influenza-like illness sentinel surveillance in one hospital in Medellin, Colombia. 2007–2012

**DOI:** 10.1111/irv.12271

**Published:** 2014-08-06

**Authors:** Ana Eugenia Arango, Sergio Jaramillo, Juan Perez, Julia S Ampuero, David Espinal, Jorge Donado, Vidal Felices, Josefina Garcia, Alberto Laguna-Torres

**Affiliations:** a Grupo Inmunovirología, Universidad de AntioquiaMedellín, Colombia; b Sede de Investigación Universitaria (SIU) Torre 2 Lab. 532, Universidad de AntioquiaMedellín, Colombia; c Hospital Pablo Tobón UribeMedellín, Colombia; d US Naval Medical Research Unit No 6, NAMRU-6Lima, Perú

**Keywords:** Influenza, Medellin, respiratory viruses, surveillance

## Abstract

**Background:**

The city of Medellin in Colombia has almost no documentation of the causes of acute respiratory infections (ARIs). As part of an ongoing collaboration, we conducted an epidemiologic surveillance for influenza and other respiratory viruses. It described the influenza strains that were circulating in the region along with their distribution over time, and performing molecular characterization to some of those strains. This will contribute to the knowledge of local and national epidemiology.

**Objectives:**

To analyze viral etiologic agents associated with influenza like illness (ILI) in participants reporting to one General hospital in Medelllin, Colombia.

**Results:**

From January 2007 to December 2012, a total of 2039 participants were enrolled. Among them, 1120 (54·9%) were male and 1364 (69%) were under the age of five. Only 124 (6%) were older than the age of 15. From all 2039 participants, 1040 samples were diagnosed by either isolation or RT-PCR. One or more respiratory viruses were found in 737 (36%) participants. Of those, 426 (57·8%) got influenza A or B. Adenoviral and parainfluenza infections represented 19·1% and 14·9% of viral infections, respectively. Influenza A was detected almost throughout the whole year except for the first quarter of 2010, right after the 2009 influenza A pandemic. Influenza B was detected in 2008, 2010, and 2012 with no pattern detected. During 2008 and 2010, both types circulated in about the same proportion. Unusually, in many months of 2012, the proportion of influenza B infections was higher than influenza A (ranging between 30% and 42%). The higher proportion of adenovirus was mainly detected in the last quarter of years 2007 and 2010. Adenoviral cases are more frequent in participants under the age of four.

**Conclusions:**

The phylogenetic analysis of influenza viruses shows that only in the case of influenza A/H1N1, the circulating strains totally coincide with the vaccine strains each year.

## Introduction

Within the etiology of acute respiratory infections (ARIs), viruses play an important role and they are the main cause of ARIs in children under the age of five^1^ and, in some cases, they also become an important cause of death.^2^ Usually, the human respiratory tract is attacked by pathogens such as influenza A and B viruses, respiratory syncytial virus (RSV), adenoviruses, and parainfluenza 1, 2, and 3 viruses,^3^ and most recently, other pathogens such as human metapneumovirus (hMPV) and rhinovirus have been detected.^4^,^5^ The RSV was reported as the main etiologic agent for ARIs in children under the age of one.^6^

Some of these agents prefer certain age groups, as RSV and rhinovirus affect mainly children under the age of one; however, they can be found at any age depending on the host's immune status. Children are very vulnerable to ARIs because of their immune system immaturity, associated with very close interpersonal contact (in day cares and schools) and many times in settings without proper protection measures such as frequent hand-washing, vaccination, and others.^7^ Chronic pathologies such as Down syndrome, cystic fibrosis, and bronchopulmonary dysplasia also favor respiratory infections.^8^,^9^

Upper respiratory infections usually present a non-specific spectrum of clinical manifestations, which hinders the correct identification of bacterial or viral infections and leads treating physicians to prescribe unnecessary antibiotics. The timely etiological diagnosis allows clinicians to properly administer drugs and implement epidemiologic measures.^10^,^11^

In Colombia, the lack of published data regarding etiologic agents and burden of respiratory disease, in particular influenza-like illness (ILI), is notable. Few studies have found the causes, frequency, and symptoms of respiratory viral infections in children, its mortality, and the potential association with peak seasons of influenza,^12^–^14^ and most of these studies have been fairly specific, developed in a short period of time and with a small sample size. A sentinel surveillance system established in Bogota and Manizales by the National Institute of Health of Colombia has shown that RSV was the most frequent agent in children under the age of five enrolled once a week in the emergency room and the daily medical consultation. Although adenovirus cases were rare, they were usually more severe.^15^,^16^

Circulating strains of RSV and hMPV from South America (including Colombian samples) were recently genetically analyzed.^17^,^18^ Also recently, the first six cases of hMPV were reported in Medellin, Colombia.^19^

As part of an ongoing collaboration among the Universidad de Antioquia in Medellin along with its Immunovirology Group, the Hospital Pablo Tobón Uribe (HPTU), and US Naval Medical Research Unit No 6 (NAMRU-6), based in Lima, Peru, an epidemiologic surveillance for influenza and other respiratory viruses in Medellin, Colombia, was conducted. It described the influenza strains that were circulating in the region along with their distribution over time and performing molecular characterization to some of those strains. This will contribute to the knowledge of local (Medellin and its metropolitan area) and national (Colombia) epidemiology, as well as that of the South American region. This epidemiologic surveillance network has previously described influenza and other respiratory virus circulation in other countries.^20^–^23^ The aim of our study was to show the clinical–epidemiologic and phylogenetic characteristics of circulating respiratory viruses in Medellin, Colombia.

## Materials and methods

### Study population and case definition

The study population included every patient with ILI or severe acute respiratory infection (SARI) cases, regardless of age, who sought attention at or was hospitalized in the HPTU facilities between January 2007 and December 2012 and agreed to participate. HPTU is a 317-bed private institution of fourth level of complexity that serves a population of around 3 000 000 persons from Medellin city and the metropolitan area. Annually, HPTU reports around 800 hospitalizations for bacterial or viral pneumonia. In 2009, there were 36 151 emergency room visits and 86 321 outpatient consultations. In addition, 1 019 907 laboratory tests and more than 700 kidney, liver, and bone marrow transplants were performed. The hospital has a 34-bed intensive care unit, which receives adults and children with major trauma and other major illnesses. During 2009, a total of nine cases of ventilator-associated pneumonia per 1000 ventilator day were reported.

Trained medical personnel were responsible for properly identifying and classifying patients with ILI or SARI. The case definition of ILI was “any person with a sudden onset of fever (oral temperature ≥38°C or axillary temperature ≥37·5°C) and either cough or sore throat.” SARI cases included the ILI case definition plus dyspnea and the need for hospitalization.”^20^,^24^ Hospitalization was defined as when the patient spent at least one night in the hospital or health center. All patients were recruited within 5 days of their initial symptoms.

Colombia is a tropical country with coasts along both oceans, located in the northern area of the Southern Cone, experiencing all climates. Medellin, with about 2 600 000 inhabitants, is the second most important city of the country and is located in a valley flanked by two ramifications of the Colombian Andean mountain range at 1475 m above sea level. Its weather is warm, at around 24°C with variations between 18 and 30°C, depending on the time of year. Throughout the year, there are two rainy seasons from March to June and from October to December and two summer seasons during the rest of months.

### Clinical findings

Signs or symptoms reported by more than 5% of participants were included in the analyses. Signs and symptoms of participants with influenza A (A), influenza B (B), adenovirus (C), RSV (D), or parainfluenza (E) infection were compared with each other looking for statistically significant differences. Each virus was identified with a letter (A, B, C, D, E), which was inserted next to the virus compared with others, when statistically significance was detected.

### Data collection and management

Data regarding gender, age, previous treatment, medical attention before enrollment, influenza vaccination status, and travels in the last 15 days were collected utilizing a case report form (CRF) from all volunteers that met the case definition and agreed to participate. In April 2008, CRF was slightly modified to better obtain data.

### Sample collection

Two types of samples were obtained for diagnosis: a nasal swab for rapid influenza test (RIT) [QuickVue Influenza Test (Quidel, San Diego, CA, USA)] and an oropharyngeal swab for RT-PCR or viral isolation. The RIT was processed at HPTU, and the results were provided to the patient after 10 minutes. Oropharyngeal swabs were placed in Universal Transport Media (Diagnostic Hybrids, Quidel Corporation) and stored at −70°C or lower as soon as possible (before 2 hours), until delivered on dry ice to NAMRU-6 in Lima, Peru, for laboratory analysis (RT-PCR and/or viral isolation for respiratory viruses).

Regular personnel trained in protocol procedures and semiannual site visits were part of the strategy to improve sampling, storage, and shipping procedures with the purpose of reducing viral death.

### Laboratory analysis

Influenza was diagnosed by either RT-PCR or isolation. From 2007 to 2010, all influenza cases were diagnosed by isolation and randomized RT-PCR was also performed. In 2009, during the pandemic, RT-PCR and isolation were performed for influenza diagnosis. From 2011 to 2012, influenza diagnoses were performed by RT-PCR in all samples and isolation was conducted in all participants under the age of five: severe cases, 10% of RT-PCR-positive samples and 20% of samples from participants older than the age of five negative by PCR. Adenovirus, parainfluenza (types 1, 2, 3, and 4), RSV, herpes virus, and hMPV were 100% diagnosed by isolation.

#### Virus isolation and detection

Patient specimens were inoculated onto three cell lines for virus isolation: Madin–Darby canine kidney (MDCK), African green monkey kidney (VERO), and rhesus kidney (LLCMK2) cells. Upon the appearance of a cytopathic effect or after 10 days of culture, cells were spotted onto microscope slides. Cell suspensions were dried and fixed into chilled acetone for 15 minutes. Immunofluorescence assay was performed using a DFA Respiratory Virus Screening and ID Kit (D3 DFA Respiratory virus Diagnostic Hybrids, Athens, OH, USA) for the detection of adenovirus, influenza A, influenza B, parainfluenza (types 1, 2, 3, and 4), and RSV. In addition, a D3 DFA HSV identification kit and a D3 IFA Enterovirus ID kit (Diagnostic Hybrids) were used for the detection of herpes virus and enterovirus, respectively, following the manufacturer's protocol as described before.^20^–^22^ VERO and LLCMK2 cell lines and metapneumovirus DFA reagent (Diagnostic Hybrids) were used for metapneumovirus detection.

#### RNA extraction and RT-PCR

Nucleic acid was extracted with the use of viral RNA kit (QIAamp, Qiagen®, Valencia, CA, USA) from 140 μl of the nasopharyngeal/oropharyngeal swab solution and was amplified in a reverse transcriptase PCR (RT-PCR). RT-PCR was performed using a SuperScript III One-Step RT-PCR System (Invitrogen, San Diego, CA, USA). A real-time PCR for influenza viruses was performed on samples as described previously.^20^

#### Phylogenetic analysis

Phylogenetic trees were generated based on the hemagglutinin (HA) gene sequence. For direct sequencing of viral nucleic acids from clinical specimens, gene fragments were amplified and sequenced with the use of Big Dye terminator cycle sequencing kit (version 3.1, Applied Biosystems, Foster City, CA, USA) on an ABI 3130XL DNA sequencer (Applied Biosystems, Foster City, CA). Nucleotide sequences of PCR products were analyzed using Sequencher software (version 4.8, Gene Codes Corporation) and then aligned with the CLUSTAL X version 2.1 software and BioEdit (version 7.2.3 -Isis Pharmaceuticals, Inc.) software to compare with influenza sequences from the GenBank database. Phylogenetic trees were developed using MEGA (version 5.2.2), the analysis was inferred using the neighbor-joining method, and the evolutionary distances were computed using the Kimura 2-parameter method. The percentage of replicate trees in which the associated taxa clustered together in the bootstrap test (1000 replicates) is shown next to the branches.

### Ethical management

This protocol was approved as less than minimal risk research by the Naval Medical Research Center (NMRC), Silver Spring, MD Institutional Review Board (IRB; NMRCD.2002.0019), and NAMRU-6 IRB (NMRCD 2011.0012) following the international regulations of human subjects research. This protocol was also approved by the Hospital Tobón Uribe local IRB. An exemption was given to perform the study using an information sheet approved and stamped by the IRB, as this was part of clinical care and routine surveillance of patients with upper respiratory infections from Hospital Tobón Uribe without any additional intervention; verbal consent was obtained from all participants after the contents of the information sheet were explained; an information sheet copy was provided to each study subject. This method of consent was accepted by the NAMRU-6 IRB, as well as by the HPTU authorities.

Country approval was given by Dirección Seccional de Salud de Antioquia and the INS (Instituto Nacional de Salud) de Colombia. Reports of findings were sent locally and also to National Health Institute in Bogotá and to HPTU.

### Statistical analysis

The clinical–epidemiologic forms were entered into a database created in Microsoft Office Access 2003. Proportions were compared using the Pearson's chi-square and Fisher's exact tests. Continuous variables with a normal distribution were compared using the independent samples *t*-test; otherwise, the Kruskal–Wallis test was applied. For all confidence intervals (CI) or statistical tests, the level of confidence was 95%. A two-tailed critical value of alpha = 0·05 was used for all statistical analyses using SPSS Statistics software version 20.0 (SPSS Inc., Chicago, IL, USA). Variables with missing data by 10% or more in forms were not analyzed.

To analyze clinical differences between the different viruses, we excluded those with coinfection and considered the five most frequent viruses diagnosed: influenza A, influenza B, adenovirus, parainfluenza, and RSV. To determine the efficacy of *in situ* influenza A and B testing, RIT results were compared with laboratory isolation or PCR results.

## Results

### General findings

From January 2007 to December 2012 a total of 2039 participants were enrolled. Among them, 1120 (54·9%) were male and 1364 (69%) were under the age of five. Only 124 (6%) were older than the age of 15 (Table 1). 85·4% of participants were from Medellin, and the others being from different areas of the Antioquia department or the rest of the country. Only 339 (16%) participants were hospitalized, and no more than 47 (2·3%) had travelled out of Medellin 15 days before the enrollment process. Influenza vaccination was reported in 133 participants (6·9%), of which 21 were positive for influenza A or B infection. Eight of 21 remembered the date of vaccination, while six of them were vaccinated more than a year before the onset of symptoms. The other two participants were vaccinated between 3 and 6 months before the onset of symptoms.

**Table 1:** Demographic and clinical findings. Hospital Tobon Uribe, 2007–2012

	Total		Positive		Negative	
	Count	%	Count	%	Count	%
Total	2039	100·0	737	100·0	1302	100·0
Gender						
Female	919	45·1	322	43·7	597	45·9
Male	1120	54·9	415	56·3	705	54·1
Age group (yrs)						
Mean ± SD	5·2 ± 8		6·4 ± 8·3		4·5 ± 7·8	
Median [range]	3 [0–76]		4 [0–70]		2 [0–76]	
N valid	2037		737		1300	
0–4	1364	66·9	404	54·8	960	73·7
5–14	551	27·0	286	38·8	265	20·4
15–29	64	3·1	21	2·8	43	3·3
30–44	40	2·0	20	2·7	20	1·5
45–59	12	0·6	3	0·4	9	0·7
60+	6	0·3	3	0·4	3	0·2
Missing	2	0·1	0	0·0	2	0·2

	*n/N*	%	*n/N*	%	*n/N*	%
Travelers in the past 15 days	47/1978	2·4	21/720	2·9	26/1258	2·1
Influenza vaccination	133/1915	6·9	48/691	6·9	85/1224	6·9
Current illness						
Medical attention before enrollment	829/1816	45·6	299/650	46·0	530/1166	45·5
Treatment						
Antibiotics	473	23·2	138	18·7	335	25·7
Antiviral	9	0·4	6	0·8	3	0·2
Antibiotics/antiviral	6	0·3	0	0·0	6	0·5
No	1462	71·7	564	76·5	898	69·0
Missing	89	4·4	29	3·9	60	4·6

	*n/N*	%	*n/N*	%	*n/N*	%	P value
Clinical findings*							
Cough	1895/2039	92·9	685/737	92·9	1210/1302	92·9	>0·05
Malaise/asthenia	1556/2038	76·3	607/737	82·4	949/1301	72·9	<0·001
Rhinorrhea	1311/2037	64·4	442/737	60·0	869/1300	66·8	0·002
Pharyngeal erythema	814/1836	44·3	336/692	48·6	478/1144	41·8	0·005
Rhonchi	531/2038	26·1	175/737	23·7	356/1301	27·4	>0·05
Wheezing	469/2038	23·0	127/737	17·2	342/1301	26·3	<0·001
Dyspnea	339/1836	18·5	72/692	10·4	267/1144	23·3	<0·001
Headache	330/2037	16·2	169/737	22·9	161/1300	12·4	<0·001
Vomit	286/1836	15·6	103/692	14·9	183/1144	16·0	>0·05
Sore throat	293/2037	14·4	133/737	18·0	160/1300	12·3	<0·001
Polypnea	263/1836	14·3	57/692	8·2	206/1144	18·0	<0·001
Conjunctival irritation	290/2038	14·2	134/737	18·2	156/1301	12·0	<0·001
Myalgias	204/2037	10·0	112/737	15·2	92/1300	7·1	<0·001
Rales	175/1836	9·5	38/692	5·5	137/1144	12·0	<0·001
Abdominal pain	166/1836	9·0	90/692	13·0	76/1144	6·6	<0·001
Adenopathy	164/1836	8·9	59/692	8·5	105/1144	9·2	<0·001
Expectoration	161/1835	8·8	53/692	7·7	108/1143	9·4	>0·05
Diarrhea	104/1836	5·7	43/692	6·2	61/1144	5·3	>0·05

* Those with <5% were excluded.

A total of 475 (23·2%) participants reported the use of antibiotics before the enrollment, and in 335 of those (71%), no viral infection was detected. Only nine participants confirmed the use of antivirals before the enrollment, but no names were registered on the forms (Table 1).

From all 2039 participants, 1040 samples were diagnosed by either isolation or RT-PCR, 857 only by isolation, and 142 only by RT-PCR. One or more respiratory viruses were found in 737 (36%) participants. Of those, 426 (57·8%) had influenza A or B. A total of 127 of 276 influenza A-positive samples were subtyped. Forty-two (15·2%) of them were A/H1N1pdm09, 12 (4·4%) were seasonal H1N1, and 73 (26·5%) were H3N2. Adenoviral and parainfluenza infections represented 19·1% and 14·9% of viral infections, respectively. In addition, 10 participants (1·3%) were coinfected. Other respiratory viruses, such as RSV, hMPV, enterovirus, and herpes virus, were also detected, ranging between 2·4% and 1·1% of the positive samples (Table 3). Influenza was the most frequent infection detected and represented 21% of all ILI cases (426/2039).

The total number of SARI patients was 146, representing 7·2% of the entire study population. Of those, 13 of 146 were influenza A positive and 2 of 146 were influenza B positive. Also, 8 (5·5%) had parainfluenza, and 2 (1·4%) RSV. Other etiologies such as hMPV and enteroviruses were <1% each.

Although all the 2039 participants were sampled, the study did not take account of the number of persons who sought attention or did not agree to participate in the HPTU during the study period.

The specificity and sensitivity of the rapid influenza test was evaluated using isolation and/or RT-PCR as the gold standard test. It was found in 2009 participants with positive cultures and/or RT-PCR. Rapid influenza A test (RIAT) specificity was 97·5% (95% CI: 96·7–98·2) and sensitivity was 63·8% (95% CI: 57·6–69·6). The positive predictive value (PPV) of the RIAT was 79·4% (95% CI: 73·2–84·6) and the negative predictive value (NPV) was 94·8% (95% CI: 93·6–95·7). Rapid influenza B test (RIBT) specificity was 99·6% (95% CI: 99·2–99·8) and sensitivity was 63·6% (95% CI: 55·3–71·1). The PPV of the RIBT was 93·2% (95% CI: 86·0–97·0) and the NPV was 97·1% (95% CI: 96·2–97·8). The RIBT has better PPV and NPV than RIAT as shown in the summary below:

**Table:** 

	Isolation and/or RT-PCR				
	Positive		Negative		
	*n*	%	*n*	%	Total
Result of the rapid influenza A test					
Positive	166	63·8	43	2·5	209
Negative	94	36·2	1706	97·5	1800
Total	260	100	1749	100	2009
Result of the rapid influenza B test					
Positive	96	63·6	7	0·4	103
Negative	55	36·4	1851	99·6	1906
Total	151	100	1858	100	2009

Missing data = 39.

### Clinical findings

Participants infected with respiratory viruses more frequently presented with malaise/asthenia, pharyngeal erythema, conjunctival irritation, sore throat, myalgia, and/or abdominal pain (*P* < 0·001) (Table 1). Symptoms such as otalgia, photofobia, eye pain, and dizziness were reported in <5% of participants.

Regarding signs and symptoms of participants identified with respiratory viral infections, headaches were significantly higher among those with influenza A (33·0%; 91/276) compared with those who reported adenovirus (17·0%; 21/141) or parainfluenza (10·0%; 11/110) infections (*P* < 0·001). In addition, myalgia was significantly higher among those with influenza A (20·7%; 57/276) and influenza B (22·0%; 33/150) infections compared with those with adenovirus (8·5%) or parainfluenza (3·6%; 4/110) infections (*P* < 0·001) (Table 2).

**Table 2:** Clinical characteristics in patients with a respiratory virus identified. Hospital Tobon Uribe, 2007–2012

	Flu A (A)		Flu B (B)		Adeno (C)		RSV (D)		Paraflu (E)	
	Count	%	Count	%	Count	%	Count	%	Count	%
Total	276	100·0	150	100·0	141	100·0	18	100·0	110	100·0
Gender										
Female	124	44·9	70	46·7	66	46·8	11	61·1	32	29·1
Male	152	55·1	80	53·3	75	53·2	7	38·9	78	70·9
Signs & symptoms*										
Cough	260/276	94·2	136/150	90·7	125/141	88·7	18/18	100·0	105/110	95·5
Rhinorrhea	159/276	57·6	83/150	55·3	94/141	66·7	17/18	94·4% A B	68/110	61·8
Pharyngeal erythema	133/263	50·6	70/142	49·3	67/132	50·8	5/15	33·3	46/100	46·0
Headache	91/276	33% C E	35/150	23·3	24/141	17·0	1/18	5·6	11/110	10·0
Sore throat	60/276	21·7	27/150	18·0	25/141	17·7	0/18	0·0	12/110	10·9
Rhonchi	59/276	21·4	37/150	24·7	38/141	27·0	6/18	33·3	25/110	22·7
Myalgia	57/276	20·7% C E	33/150	22·0 C E	12/141	8·5	2/18	11·1	4/110	3·6
Conjunctival irritation	54/276	19·6	27/150	18·0	22/141	15·6	3/18	16·7	16/110	14·5
Vomit	39/263	14·8	10/142	7·0	29/132	22·0 B	2/15	13·3	18/100	18·0
Wheezing	39/276	14·1	24/150	16·0	24/141	17·0	7/18	38·9	27/110	24·5
Abdominal pain	33/263	12·5	19/142	13·4	22/132	16·7	0/15	0·0	8/100	8·0
Dyspnea	25/263	9·5	6/142	4·2	15/132	11·4	5/15	33·3% B	19/100	19·0% B
Expectoration	24/263	9·1	7/142	4·9	8/132	6·1	2/15	13·3	9/100	9·0
Adenopathy	23/263	8·7	9/142	6·3	17/132	12·9	2/15	13·3	6/100	6·0
Diarrhea	21/263	8·0	6/142	4·2	12/132	9·1	0/15	0·0	3/100	3·0
Tachypnea	15/263	5·7	6/142	4·2	13/132	9·8	6/15	40·0 A B C	13/100	13·0
Rales	13/263	4·9	3/142	2·1	7/132	5·3	3/15	20·0	11/100	11·0

Each virus was identified with a letter (A, B, C, D, E), which was inserted next to the virus compared to others, when statistically significance was detected.

* Excluded: coinfections.

It was recently reported that incidents of tachypnea were much higher in patients with RSV infections (40·0%; 6/15) compared with those with influenza A (5·7%; 12/263), influenza B (4·2%; 6/142), and adenovirus (9·8%; 13/132) infections (*P* < 0·001). Furthermore, Table 2 shows that rhinorrhea was significantly greater among those with RSV infections (94·4%; 17/18) compared with those who had influenza A (57·6%; 159/276) and influenza B (55·3%; 83/150) infections (*P* < 0·001).

Dyspnea was also significantly higher (*P* < 0·001) among those with parainfluenza (19%; 19/100) or RSV (33·3%; 5/15) infections compared with those who had influenza B infections alone. We found no statistical significance when comparing symptoms of sore throat, ronchi, conjunctival irritation, and wheezing in patients infected with any of the five most frequently identified viruses in this study.

An analysis of the manuscript was performed in the viral-positive SARI participants, and no differences were found with the total population as shown in Table 2.

### Viral distribution by time and age: Seasonality

**Figure 1:**
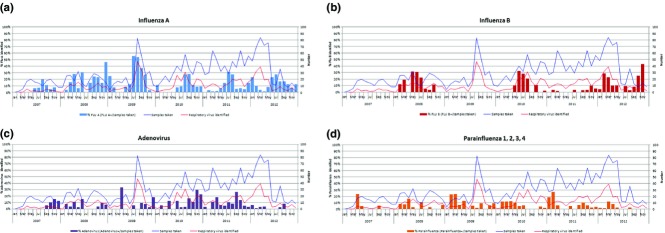
Proportion of samples with a respiratory virus identified by month. Hospital Tobon Uribe 2007–2012. During all 6 years of study, influenza A virus was circulated almost throughout the year. Only three clearly defined influenza B circulation periods were observed, in 2008, 2010, and 2012. (A) Proportion of influenza A samples identified. (B) Proportion of influenza B samples identified. (C) Proportion of adenovirus samples identified. (D) Proportion of parainfluenza 1, 2, 3, and 4 samples identified.

The most frequent respiratory viruses identified in Hospital Tobon Uribe from 2007 to 2012 were influenza A, influenza B, adenovirus, and parainfluenza (Table 3). The temporal distribution of the proportion of positive viruses by isolation or PCR over the number of samples taken and the number of respiratory viruses identified is shown in Figure 1.

**Table 3:** Viral etiology by year. Hospital Tobon Uribe 2007–2012

	Total		Year					
	Count	%	2007	2008	2009	2010	2011	2012
Total	2039	100·0	143	243	304	380	518	451
Positive results by PCR or isolation	737	36·1	25	105	134	158	153	162
Influenza A (FLU A)	276	37·4	8	42	88	44	49	45
H1N1pdm09	42	15·2	0	0	2	3	35	2
H1N1 seasonal	12	4·3	0	12	0	0	0	0
H3N2	73	26·4	8	1	1	6	14	43
Influenza B (FLU B)	150	20·4	0	29	0	41	13	67
Adenovirus	141	19·1	6	12	16	44	51	12
Parainfluenza (paraflu)	110	14·9	6	11	22	17	33	21
Respiratory syncytial virus (RSV)	18	2·4	3	0	3	5	2	5
Metapneumovirus (hMPV)	15	2·0	0	1	1	4	1	8
Enterovirus	9	1·2	1	4	2	1	1	0
Herpes virus (HSV)	8	1·1	1	2	1	0	0	4
Adenovirus – paraflu 2	1	0·1	0	0	0	0	1	0
Adenovirus – enterovirus	2	0·3	0	0	0	0	2	0
Adenovirus – flu A	1	0·1	0	1	0	0	0	0
Adenovirus – flu B	2	0·3	0	0	0	2	0	0
Adenovirus – hMPV	1	0·1	0	1	0	0	0	0
Adenovirus – paraflu 1	1	0·1	0	1	0	0	0	0
Echovirus – flu A	1	0·1	0	1	0	0	0	0
Enterovirus – flu A	1	0·1	0	0	1	0	0	0
Negative	1302	63·9	118	138	170	222	365	289

Influenza A was detected almost throughout the year except for the first quarter of 2010, just after the 2009 influenza A pandemic. Overall, the proportion of influenza A infections (FLUA+/samples taken) increases every year after June (Figure 1A). The high proportion of influenza A cases was detected in July 2009 (56%). Influenza B was detected in 2008, 2010, and 2012 with no pattern detected. During 2008 and 2010, both types circulated in about the same proportion. Unusually, in many months of 2012, the proportion of influenza B infections was higher than influenza A (ranging between 30% and 42%) (Figure 1B). No trends were found in the temporal distribution of parainfluenza viruses (Figure 1D).

**Figure 2:**
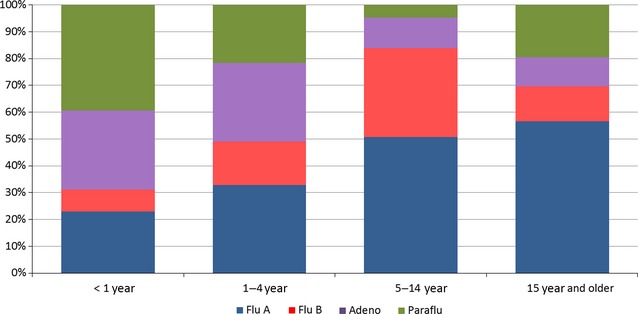
Viral detection by age group. Hospital Tobon Uribe, 2007–2012.

The higher proportion of adenovirus was mainly detected in the last quarters of years 2007 and 2010 (Figure 1C). Adenoviral cases are more frequent in participants under the age of four (Figure 2).

Participants under the age of one mainly had parainfluenza and adenovirus infections, but participants older than the age of 15 were mainly infected with influenza A or B (Figure 2).

A lack of seasonality for any of the respiratory pathogens was found in this study.

### Phylogenetic analysis of the influenza isolates

Genetic analyses in Figures 5 show that in all three cases, influenza circulating strains have changed over the years as in other countries in Latin America as previously shown.^20^,^22^,^23^ In many years, the isolated A/H3N2 and B subtype strains did not coincide with the recommended vaccine, although it did for A/H1N1 and A/H1N1pdm09 strains.

**Figure 3:**
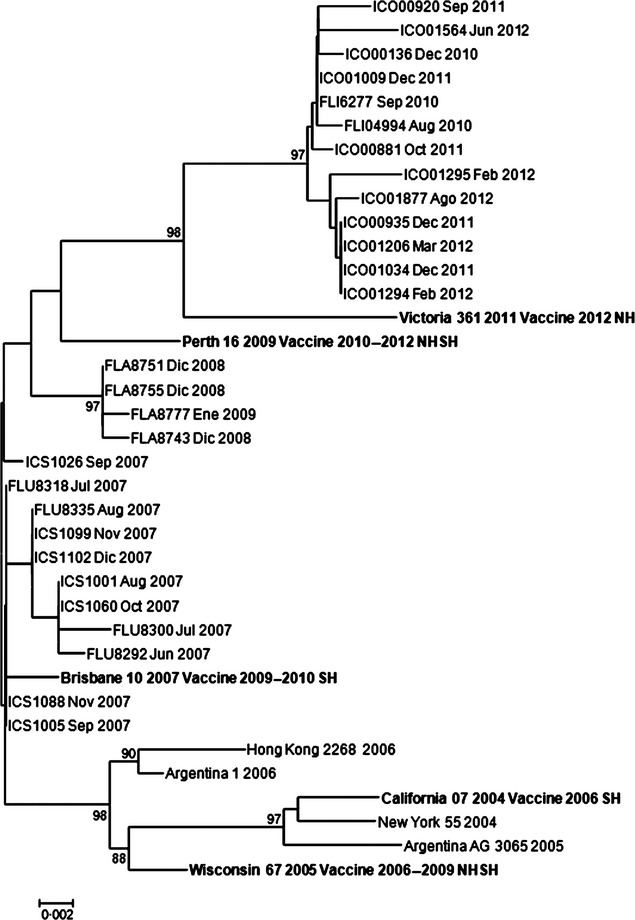
Phylogenetic analysis. Influenza A/H3N2 strains. Hospital Tobon Uribe, 2007–2012. Some 2008 and one 2009 strains do not group with the recommended vaccine strain for those years. On the other hand, 2007 and 2010–2012 strains grouped to the selected vaccine strain.

**Figure 4:**
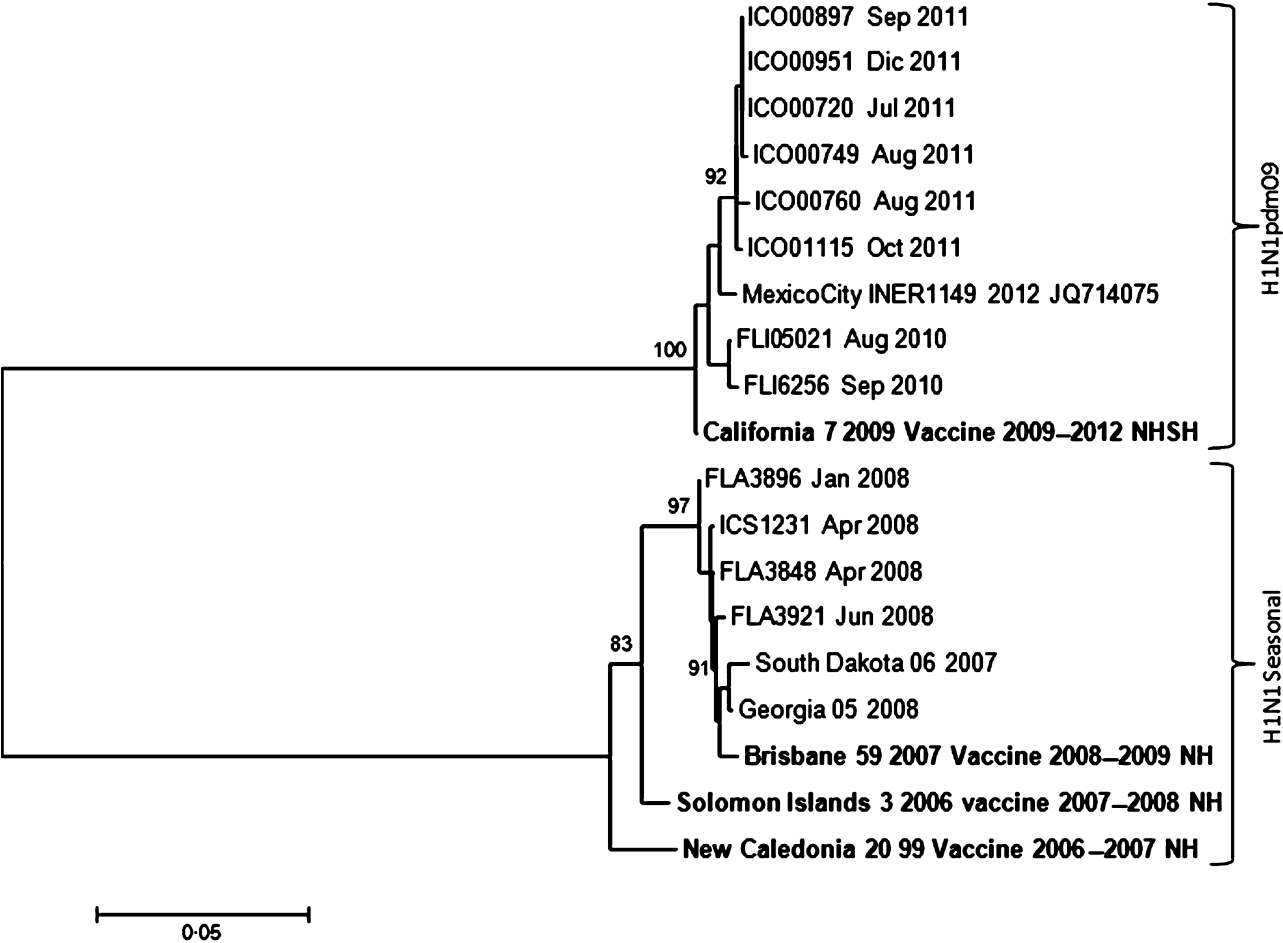
Phylogenetic analysis. Influenza A/H1N1 strains. Hospital Tobon Uribe, 2007–2012. The circulating strains grouped into two clades and coincide with the vaccine strains each year.

**Figure 5:**
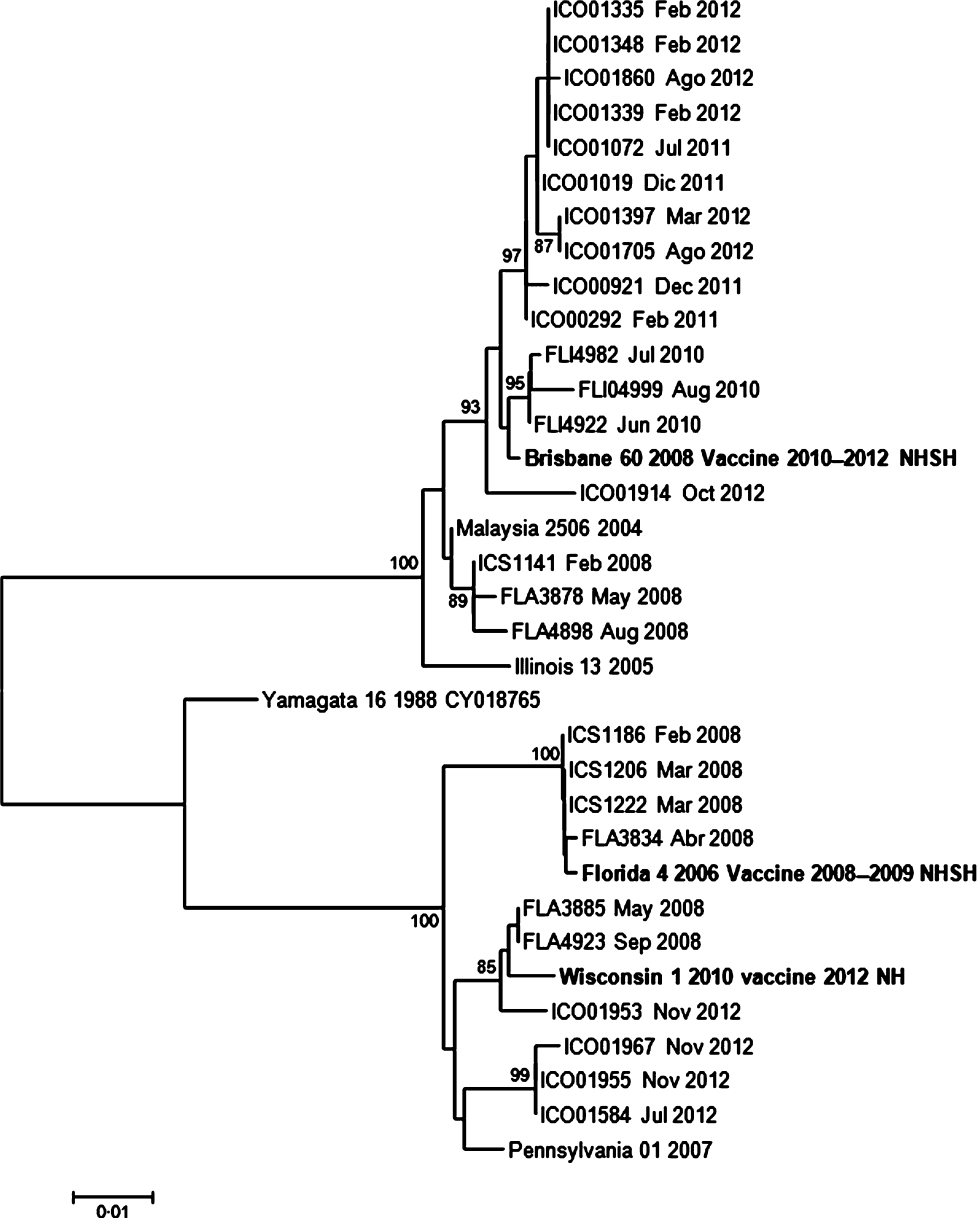
Phylogenetic analysis. Influenza B strains Hospital Tobon Uribe, 2007–2012. Some strains analyzed from 2010 to 2012 grouped to Brisbane/60/2008 strain, which corresponds to the period vaccine strain. Some samples from 2012 grouped to Wisconsin and Florida, but those were not the vaccine selected strains for that year. Previous strains from 2008 grouped to Wisconsin, Florida, and Brisbane strains.

Influenza A/H3N2 isolates from Medellin revealed three different genotypes (i) Brisbane/10/2007-like (ii) Perth/16/2009-like, and (iii) Victoria/361/2011-like (Figure 3). Samples circulating in 2007 grouped within the A/Brisbane/10/2007 strain (recommended vaccine for 2008/2009/2010 SH). The latter also includes the recommended 2006 vaccine strain for the southern hemisphere, California/07/2004- and the Wisconsin 67/2005-like strain recommended for 2006–2007 NH and for 2007 SH. (Figure 3). On the other hand, we found some samples circulating in 2008–2009 similar to the Perth/16/2009 strain, and finally, samples circulating in 2010–2012 were similar to the Victoria/361/2011-like lineage.

Influenza A/H1N1 strains (Figure 4) grouped into two clear clades, one with seasonal H1N1 strains before 2009, similar to the Brisbane/59/2007 vaccine strain, and the other corresponding to the pandemic lineage H1N1pdm09 similar to the California/07/2009-like strain.

Finally, for the influenza B strains in circulation, Figure 5 shows that there have been different strains cocirculating in Medellin. Influenza B strains (Figure 5) grouped into three clades: similar to Brisbane/60/2008, Wisconsin/01/2010, and Florida/04/2006 strains. The latter was the recommended vaccine for 2008–2010 SH. During 2012, we also detected some circulating strains not related to the southern hemisphere 2012 vaccine.

## Discussion

Prior to this study, there was a paucity of published data about respiratory infections, in Medellin. Other comparable studies use tests such as immunofluorescence or immunoenzymatic assays, whereas this study uses viral cultures and/or RT-PCR for diagnostic confirmation. The duration of the study and the significant number of samples collected will help local physicians and epidemiologists to understand the clinical and epidemiologic characteristics of viral respiratory infections in a high complexity hospital of Medellin. However, as this study was conducted as a permanent surveillance of ILIs in one hospital, data cannot be generalized to the Colombian population, including that of Medellin.

Usually in Colombia, viral ARI diagnosis is based upon clinical data and immunofluorescence or immunoenzymatic assays for respiratory viruses. The Instituto Nacional de Salud of Bogota is the only one that uses RT-PCR for influenza diagnosis. This study found viral agents undetected before such as human metapneumovirus, a viral pathogen first reported in Colombia by our group.^19^ Using three different cell lines for isolation addressed the potential lack of local viral isolation capabilities also. This should contribute to broadening the physician's view on viruses associated with respiratory tract infections.

Clinical symptoms normally do not allow for distinguishing the agents causing ARIs; however, some clinical manifestations are more characteristic in certain types of viruses, such as bronchiolitis and RSV infection.^25^ Cough, malaise, and rhinorrhea are the main symptoms among ARI patients; nevertheless, these symptoms are not characteristic of a certain pathogen agent. Some clinical manifestations are more frequent in children and not so common among adults, such as manifestations in the digestive tract (diarrhea, vomits, and abdominal pain);^26^ in our case, vomits (15·6%) were the most frequent digestive manifestation among children.

During this surveillance system, samples were collected throughout the year as a reflection of the occurrence of medical visits based on respiratory diseases in general Medellin population, although there was a peak of visits in 2009, very likely because of the influenza A/pH1N1 pandemic emergence in April 2009. Besides, in 2009 and 2010, the winter season caused by La Niña phenomenon in Colombia had major rainfalls compared with reports from other years.^27^

This study focused on ILI cases, and although 69% of our population was under the age of five, only a small number of RSV cases (2·4%) were reported. RSV is an extremely labile virus,^28^ and even though our samples were kept frozen at −70°C from collection to processing in Lima, Peru, this aspect plus time-related issues between collection and processing likely resulted in the small number of cases in this report. It has also been demonstrated previously that immunofluorescence on cultured cells is not the best technique for RSV detection.^29^–^31^ In Argentina, for instance, immunofluorescence was used to identify RSV in a pediatric population (27%), because it is one of the most common viruses found among children.^32^ Even in young children with bronchiolitis, RSV was the most common virus, detected in 77% of patients.^33^

This study clearly shows (Figure 2) that during the first year of life, non-influenza viral agents are predominant (approximately 70%), which changes as one gets older. Between one and 4 years of age, both influenza and non-influenza cases represent 50%. From five years to fourteen, and during young adulthood, the percentage of influenza cases predominate (70%). For all other age ranges, non-influenza viruses again become more frequent, as is the case of RSV and parainfluenza.

As far as influenza A and B viruses, though detected at a very early age, most cases occurred in children aged between 5 and 10, as published elsewhere,^34^ whereby school children are the main target of the viral infection and disseminators at home and school. In our study, 276 (37·4%) of positive cases were influenza A and 150 (20·4%) were influenza B, which highlights the importance of these viruses in the genesis of local respiratory infections.

The finding of a most affected population (school children) would also be useful to implement epidemiologic measures in the population, that is, intensifying vaccination in these age groups to try to control epidemics, without neglecting higher risk groups. Following influenza, adenovirus was found in 141 (19·1%) of positive cases, and although they were present throughout the period of the study, there was a significant increase in cases between September and December only in 2007 and 2010. During the study period, the case distribution fails to present defined seasonality (Figure 1). In 2010, this overlapped an adenovirus outbreak reported in Bogota by health authorities during the same period (unpublished). Adenoviruses became a very important cause of ILI in our study, especially among children under the age of five, as published elsewhere.^35^

During all 6 years of study, influenza A virus circulated almost throughout the year. Only three clearly defined influenza B circulation periods were observed, in 2008, 2010, and 2012. This suggests that influenza B has a biannual circulation in the metropolitan area of Medellin. Most cases occurred between April and August toward the end of the first rainy season and during dry season or summer. In other publications of the Latin American region such as Ecuador, Venezuela, and Peru, this biannual pattern is not clearly defined and influenza A and B viruses can be found in almost all years reported.^20^,^22^,^23^

The MoH in Colombia has decided to use the southern hemisphere influenza vaccine, considering the circulation of strains around the region. So, the influenza vaccination campaign starts at the beginning of each year and extends through the rest of it depending on the vaccine availability. Privilege is granted to populations at risk, such as subjects older than the age of 60, pregnant women, small children, and subjects with a basal illness.

As far as frequency, after influenza A and B and adenovirus, parainfluenza viruses follow and circulate all year round, but if virus types are analyzed as a whole, most of them appear in the first semester of the year and overlap the first winter season and the most affected group is that of children under the age of five, as reported elsewhere.^34^ Adenovirus being most associated with a coinfection is also interesting; however, in other studies,^22^,^35^,^36^ other viruses were more frequent found in coinfections, reason for which this piece of information may not be epidemiologically significant, yet it may be clinically important if we consider adenoviruses may produce severe clinical manifestations that can worsen those caused by another coinfection-associated virus; however, several studies do not report any difference in severity. In this study, it was associated with influenza A, influenza B, parainfluenza 1, HSV, and hMPV in the coinfection. The presence of coinfections with one or more viral agents has been reported quite frequently in the literature, but the clinical and epidemiologic significance is yet to be determined. These coinfections can benefit from the fact that patients may have basal diseases that favor both viral colonization and bacterial colonization. When several viral agents circulate simultaneously, the following can happen: an individual can get infected by two or more of them, though that it is not what “normally” occurs. A question that also comes up in the case of co infections is which of the two or more viral agents is producing the clinical manifestations mainly: Is it adenovirus or the associated virus? Yet, there are asymptomatic or mild cases among viral respiratory infections, and thus, it is difficult to elucidate what the contribution of each virus to the clinical manifestation is.

Parainfluenza viruses 1–3 are human pathogens that are more frequent than parainfluenza 4, as reported by the literature. We only found two cases of parainfluenza four during the study. Other parainfluenza viruses circulated sporadically, simultaneously, and in low proportion with respect to the cases. However, all virus types were detected mostly in children under the age of five, especially in those under the age of two. It is known that other viral agents may affect the respiratory tract although this is not its main target organ. Such is the case of enterovirus and herpes simplex, but as found in our study when they were the only isolated agent, we should think they are the ones causing ILI. Nevertheless, as expected, its frequency is very low and other characteristics of the patient may have influenced to favor this infection.

Medellin is a city with tropical climate where the seasons are not clearly defined; rather, there are seasons of rainfall and lack of rainfall, which locally are called winter and summer. We definitely found lack of clear seasonal trends for any of the respiratory pathogens.

One objective of this study was to identify the circulating influenza strains helping the evaluation of current vaccine components and inform of the composition of the future vaccine. Influenza vaccination was massive in many countries after the 2009 pandemic, and in Colombia, the southern vaccine was mainly used. However, our phylogenetic trees show that for serotypes B and A/H3N2, different strains have circulated simultaneously in Colombia, and both, northern hemisphere and southern hemisphere strains, were observed. This situation is not seen with subtype A/H1N1pdm09 because since its appearance in 2009, the strain has remained similar and changes have happened worldwide quite rapidly. These findings raise the importance of a closer observation of circulating strains to select the most effective vaccine for this country.

More importantly, in the case of A/H3N2 strains, our genetic analyses revealed that some 2008 and 2009 strains found by our system do not group with the recommended vaccine strain for those years and suggest that the vaccine could not have been protective against those circulating strains. We found isolations from 2010 to 2012 grouped with the selected vaccine strains Perth/16/2009 and Victoria 361/2011. Then, patients who got any of the influenza strains analyzed in 2010–2011 could have been protected by the yearly vaccine. On the other hand, some strains from 2008 and one from 2009 grouped to Perth/16/2009 as well, but Perth was not the selected vaccine those years. Many strains from 2007 grouped to Brisbane/10/2007 suggesting that in 2007 the vaccine was protective. It could be said that the California strain from 2007 was replaced by the Perth strain in 2008 and this trend is ongoing.

In contrast to the genetic diversity observed on A/H3N2 viruses, the phylogenetic analysis of some influenza strains isolated during the study period showed that A/pH1N1 strains grouped to both California 7/09 and 2009 pandemic Mexico strains. Other strains from 2007 to 2008 grouped to the seasonal strain, Brisbane, as expected. This suggests that the vaccine was protective against the circulating strains in all those years. Our system only found 02 samples positive for influenza H1N1pdm09 in 2012. As far as influenza B strains, the phylogenetic analysis showed that samples analyzed from 2010 to 2012 grouped to Brisbane/60/2008 strain, which corresponds to the period vaccine strain. Previous strains from 2008 grouped to Wisconsin, Florida, and Brisbane strains. The vaccination campaign that year offered Florida 4/2006 strain in the SH vaccine. Our system did not find influenza B cases in 2007 and 2009. Some samples from 2012 grouped to Wisconsin and Florida as well, but those were not the vaccine selected strains for that year.

The phylogenetic analysis of influenza viruses shows that strains in circulation are similar to the ones detected in the northern part of the hemisphere and the comparison to the northern hemisphere and southern hemisphere vaccine strains shows that only in the case of influenza A/H1N1, the circulating strains coincide with the vaccine strains each year.

One of the limitations of this study is related to RSV circulation in HPTU. A lack of RSV circulation among young children was found, with a very low percentage compared with what has been published in the literature, where RSV was reported as the primary viral agent in children under the age of one. We believe that our results present low numbers of RSV-positive samples because RSV was diagnosed by isolation. We did not perform immunofluorescence from nasopharyngeal secretions directly, but from cultured cells with cytopathic effect. Also, RSV viability is known to be reduced severely when kept out of the −70 C range even for a short period of time, and it is possible that a lapse of time occurred between sample removal and processing, which could have decreased the number of RSV-positive cases. It is also important to mention that the cell lines used in this study work especially well for influenza virus isolation; therefore, although we were able to isolate some RSV, the true number of positive samples may have been missed or gone undiagnosed due to the cell line chosen for isolation. Definitely, RSV circulation in the pediatric population going to HPTU is not as low as this study showed. It would be necessary to develop a specific research to evaluate the real incidence of RSV in HPTU. Another study limitation was the symptomatology reported by the child caregiver, though forwarded by a physician. In addition, sampling was non-probabilistic of consecutive cases of patients seen in both the outpatient area and the emergency room of a high complexity hospital of Medellin; hence, internal validity of these results is meant for the hospital only. The low number of SARI participants does not represent a high impact in the results.

Nevertheless, our passive surveillance study was able to demonstrate the clinical and epidemiologic manifestations of viral respiratory infections in the initial patient visit and the phylogenetic distribution of influenza cases over time, elements that contribute to the relatively sparse description of ILI in a high complexity hospital of Medellin. Future longitudinal studies will help better characterize the clinical course of these patients in Medellin.

This study has allowed us to have a more realistic view of what happens with influenza and other respiratory viruses in the metropolitan area of Medellin. We can assure all studied viruses circulate locally and this contributes to broadening the primary and specialized physicians' observation that we should consider a whole spectrum of respiratory viral agents to implement adequate surveillance measures and a pathology-based treatment.

## Disclaimers

The views expressed in this article are those of the authors and do not necessarily reflect the official policy or position of the Department of the Navy, Department of Defense, nor the U.S. Government. The study protocol was approved by the Instituto Nacional de Salud de Colombia of Ministry of Health of Colombia, Health Direction of Antioquia, The Universidad de Antioquia, Medellin, and the Naval Medical Research Center Institutional Review Board (Protocol NMRCD.2002.0019) in compliance with all applicable Federal Regulations governing the protection of human subjects.

## Copyright statement

Authors V. Alberto Laguna, Sonia Ampuero. Josefina Garcia, Juan Perez, and Vidal Felices are employees of the U.S. Government. This work was prepared as part of their official duties. Title 17 U.S.C. § 105 provides that “Copyright protection under this title is not available for any work of the United States Government.” Title 17 U.S.C. § 101 defines a U.S. Government work as a work prepared by a military service members or employees of the U.S. Government as part of those person's official duties.

## References

[1] Luksic I, Kearns PK, Scott F, Rudan I, Campbell H, Nair H (2013). Viral etiology of hospitalized acute lower respiratory infections in children under 5 years of age – a systematic review and meta-analysis. Croat Med J.

[2] Karaivanova GM (1995). Viral respiratory infections and their role as public health problem in tropical countries (review). Afr J Med Med Sci.

[3] Heymann PW, Carper HT, Murphy DD (2004). Viral infections in relation to age, atopy, and season of admission among children hospitalized for wheezing. J Allergy Clin Immunol.

[4] Stockton J, Stephenson I, Fleming D, Zambon M (2002). Human metapneumovirus as a cause of community-acquired respiratory illness. Emerg Infect Dis.

[5] Hayden FG (2004). Rhinovirus and the lower respiratory tract. Rev Med Virol.

[6] Hall CB, Weinberg GA, Blumkin AK (2013). Respiratory syncytial virus-associated hospitalizations among children less than 24 months of age. Pediatrics.

[7] Munoz FM (2002). The impact of influenza in children. Semin Pediatr Infect Dis.

[8] Whiteson KL, Bailey B, Bergkessel M (2014). The upper respiratory tract as a microbial source for pulmonary infections in Cystic Fibrosis: parallels from Island Biogeography. Am J Respir Crit Care Med.

[9] Jama-Alol KA, Moore HC, Jacoby P, Bower C, Lehmann D (2014). Morbidity due to acute lower respiratory infection in children with birth defects: a total population-based linked data study. BMC Pediatr.

[10] Friedman BC, Schwabe-Warf D, Goldman R (2011). Reducing inappropriate antibiotic use among children with influenza infection. Can Fam Physician.

[11] Vergidis P, Hamer DH, Meydani SN, Dallal GE, Barlam TF (2011). Patterns of antimicrobial use for respiratory tract infections in older residents of long-term care facilities. J Am Geriatr Soc.

[12] Bedoya VI, Abad V, Trujillo H (1996). Frequency of respiratory syncytial virus in hospitalized infants with lower acute respiratory tract infection in Colombia. Pediatr Infect Dis J.

[13] Ramirez AP, Mendoza AR, Montoya JM (2009). Mortality associated with peak seasons of influenza virus circulation in Bogota, Colombia, 1997-2005. Rev Panam Salud Publica.

[14] Herrera D, de la Hoz F, Velandia M (2008). Severe respiratory disease and its relationship with respiratory viruses in Colombia. Int J Infect Dis.

[15] Herrera-Rodriguez DH, de la Hoz F, Marino C, Ramirez E, Lopez JD, Velez C (2007). Adenovirus in children under five years of age. Circulation patterns and clinical and epidemiological characteristics in Colombia, 1997-2003. Rev Salud Publica (Bogota).

[16] Herrera-Rodriguez DH, de la Hoz F, Marino C, Ramirez E (2007). Respiratory virus in children aged less than 10 years old suffering from respiratory infection in the Hospital Militar Central in Bogota from 2000-2001. Rev Salud Publica (Bogota).

[17] Garcia J, Sovero M, Kochel T (2012). Human metapneumovirus strains circulating in Latin America. Arch Virol.

[18] Sovero M, Garcia J, Kochel T (2011). Circulating strains of human respiratory syncytial virus in central and south America. PLoS ONE.

[19] Espinal DA, Hurtado IC, Arango AE, Garcia J, Laguna-Torres VA, Jaramillo S (2012). Human metapneumovirus in children: first cases in Colombia. Biomedica.

[20] Laguna-Torres VA, Gomez J, Ocana V (2009). Influenza-like illness sentinel surveillance in Peru. PLoS ONE.

[21] Laguna-Torres VA, Sanchez-Largaespada JF, Lorenzana I (2011). Influenza and other respiratory viruses in three Central American countries. Influenza Other Respi Viruses.

[22] Douce RW, Aleman W, Chicaiza-Ayala W (2011). Sentinel surveillance of influenza-like-illness in two cities of the tropical country of Ecuador: 2006-2010. PLoS ONE.

[23] Comach G, Teneza-Mora N, Kochel TJ (2012). Sentinel surveillance of influenza-like illness in two hospitals in Maracay, Venezuela: 2006-2010. PLoS ONE.

[24] PAHO-CDC (2006). Generic Protocol for Influenza Surveillance.

[25] Miller EK, Gebretsadik T, Carroll KN (2013). Viral etiologies of infant bronchiolitis, croup and upper respiratory illness during 4 consecutive years. Pediatr Infect Dis J.

[26] Rodrigo C, Mendez M (2012). Clinical and laboratory diagnosis of influenza. Hum Vaccin Immunother.

[27] Poveda G, Rojas W, Quinones ML (2001). Coupling between annual and ENSO timescales in the malaria-climate association in Colombia. Environ Health Perspect.

[28] Walsh EE, Falsey AR, Swinburne IA, Formica MA (2001). Reverse transcription polymerase chain reaction (RT-PCR) for diagnosis of respiratory syncytial virus infection in adults: use of a single-tube “hanging droplet” nested PCR. J Med Virol.

[29] Syrmis MW, Whiley DM, Thomas M (2004). A sensitive, specific, and cost-effective multiplex reverse transcriptase-PCR assay for the detection of seven common respiratory viruses in respiratory samples. J Mol Diagn.

[30] Gueudin M, Vabret A, Petitjean J, Gouarin S, Brouard J, Freymuth F (2003). Quantitation of respiratory syncytial virus RNA in nasal aspirates of children by real-time RT-PCR assay. J Virol Methods.

[31] Kuypers J, Campbell AP, Cent A, Corey L, Boeckh M (2009). Comparison of conventional and molecular detection of respiratory viruses in hematopoietic cell transplant recipients. Transpl Infect Dis.

[32] Marcone DN, Ellis A, Videla C (2013). Viral etiology of acute respiratory infections in hospitalized and outpatient children in Buenos Aires, Argentina. Pediatr Infect Dis J.

[33] Stempel HE, Martin ET, Kuypers J, Englund JA, Zerr DM (2009). Multiple viral respiratory pathogens in children with bronchiolitis. Acta Paediatr.

[34] Taubenberger JK, Morens DM (2008). The pathology of influenza virus infections. Annu Rev Pathol.

[35] Ampuero JS, Ocana V, Gomez J (2012). Adenovirus respiratory tract infections in Peru. PLoS ONE.

[36] Bicer S, Giray T, Col D (2013). Virological and clinical characterizations of respiratory infections in hospitalized children. Ital J Pediatr.

